# Prognostic Implications of a Second Peak of High-Sensitivity Troponin T After Myocardial Infarction

**DOI:** 10.3389/fcvm.2021.780198

**Published:** 2022-01-31

**Authors:** Tau S. Hartikainen, Alina Goßling, Nils A. Sörensen, Jonas Lehmacher, Johannes T. Neumann, Stefan Blankenberg, Dirk Westermann

**Affiliations:** ^1^Department of Cardiology, University Heart and Vascular Center Hamburg, Hamburg, Germany; ^2^German Center for Cardiovascular Research (DZHK), Partner Site Hamburg/Kiel/Lübeck, Hamburg, Germany; ^3^Department of Epidemiology and Preventive Medicine, School of Public Health and Preventive Medicine, Monash University, Melbourne, VIC, Australia

**Keywords:** myocardial infarction, biomarker, acute coronary syndrome, troponin, kinetics, second peak

## Abstract

**Background:**

After an acute myocardial infarction (MI), repeated measurement of cardiac biomarkers is commonly performed, although not recommended in current guidelines. There is only limited data on the kinetics of troponin in this phase. For high-sensitivity cardiac troponin T (hs-cTnT), but not high-sensitivity cardiac troponin I (hs-cTnI), late increases in terms of a second peak have been described. Their impact on the prognosis of patients with MI remains unclear.

**Methods:**

We included 2,305 patients presenting to the emergency department with symptoms suggestive of MI. Five hundred and seven were diagnosed with MI. Hs-cTnT, creatine kinase (CK) and the MB fraction of CK (CK-MB) were measured at admission, after 1 and 3 h and thereafter as indicated by the treating physician. A mixed-model approach was applied for modeling the biomarker kinetics. All patients were followed up to assess a composite endpoint of mortality, recurrent MI, revascularization and rehospitalization and to investigate the effect of a second hs-cTnT peak on prognosis.

**Results:**

Out of 507 patients with MI, 192 had a sufficient amount of hs-cTnT measurements after the index MI. In 111 (57.8%) patients a second hs-cTnT peak was found after 4.48 days. For CK and CK-MB a second peak could not be identified. Regarding the composite endpoint there was no significant difference between patients with and without a second hs-cTnT peak.

**Conclusion:**

In our analyses, a second peak of hs-cTnT after an acute MI was common, but not associated with poorer outcome. Thus, the clinical value of hs-cTnT for monitoring myocardial ischemia might be limited in this phase and other biomarkers might be more suitable.

**Trial Registration:** www.ClinicalTrials.gov, identifier: NCT02355457, Date of registration: February 4, 2015.

## Introduction

High sensitivity cardiac troponin (hs-cTn) has evolved as being the gold-standard biomarker for diagnosing myocardial injury and infarction (MI) (1, 2). Even though not recommended in current guidelines, repeated measurement of hs-cTn is commonly performed in clinical practice in the first days after MI. However, there is only limited data on the kinetics of hs-cTn after an acute MI and their consecutive clinical and prognostic implications. For hs-cTnT, but hs-cTnI, late increases in terms of a second peak several days after the index event have been observed (3). We aimed to investigate the prognostic value of late hs-cTnT increases after an acute MI in a large, contemporary cohort study.

## Materials and Methods

For this analysis we used data from the Biomarkers in Acute Cardiac Care study population (4). Briefly, we prospectively recruited patients presenting to the emergency department with symptoms suggestive of MI. Hs-cTnT (Elecsys; Roche Diagnostics), creatine kinase (CK) and the MB fraction of CK (CK-MB) were measured at admission, after 1 and 3 h and thereafter as indicated by the treating physician. Measurements until the 10th day after admission were included. A second peak of hs-cTnT was defined as an increase of at least 15% after the concentrations had already decreased after the first maximum troponin value. Patients without a second peak and <3 measurements between day 1 and 5 were excluded due to possible missing of an existing second peak. The final diagnosis was adjudicated by two physicians separately in a blinded fashion based on all available clinical findings and according to the Third Universal Definition of MI (5). A follow-up was performed up to 4 years. Cox regression analyses were conducted for a composite endpoint of all-cause mortality, recurrent MI, revascularization and rehospitalization. Event rates were calculated using the Kaplan-Meier estimator. For modeling the hs-cTnT kinetics a mixed-model approach was used.

## Results

**Figure 1:**
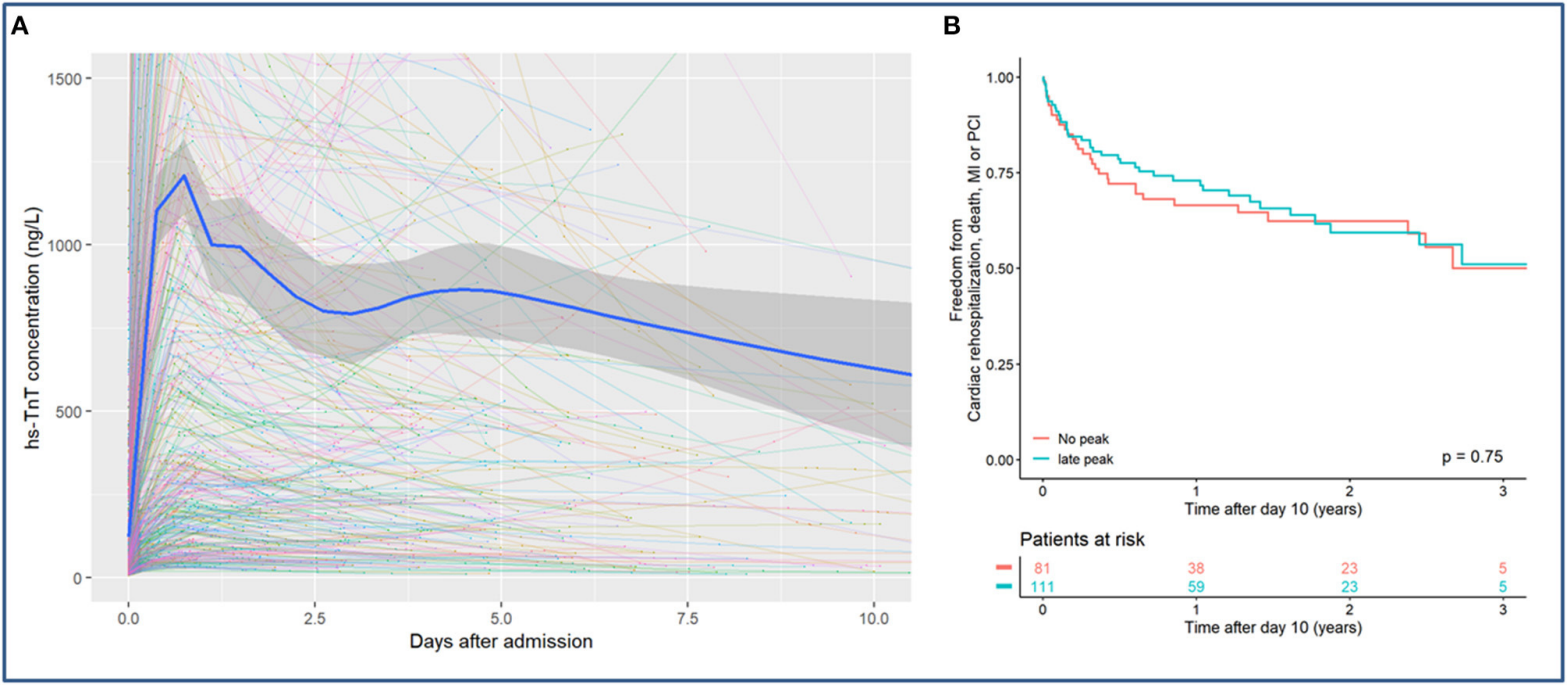
**(A)** Kinetics of hs-cTnT during the first 10 days after myocardial infarction presented using mixed-model statistics. A second hs-cTnT peak can be detected after 4.48 days after the index event. **(B)** Kaplan-Meier curve for the combined endpoint of all-cause mortality, rehospitalization, MI or PCI for patients with and without a second hs-cTnT peak.

Among 2,305 patients presenting with suspected MI, 507 (22.0%) were diagnosed as having MI. Out of these, 365 (72%) were patients with type 1 MI, 140 (27.6%) patients with type 2 MI and 2 (0.4%) patients with type 4 MI. Three hundred and eleven of all patients with MI were excluded from the analyses due to an insufficient number of available biomarker results after MI. Out of 192 remaining patients, 111 (57.8%) patients presented a second hs-cTnT peak, mostly between day 2 and 5 after the index event (Figure 1). The median troponin concentration at the first peak was 1,213 ng/L (interquartile range (IQR) 1,107–1,319 ng/L) and at the second peak 866 ng/L (IQR 727–1,005 ng/L), thus the second peak was 28.6% (IQR 23.8-34.3%) lower as compared to the first peak. In 81 patients (42.2%) a second peak could not be identified. There were no significant differences in the baseline characteristics between patients with and without a second hs-cTnT peak (Table 1). Angiography for the index event was performed in 97.5% in patients without and 92.8% with a second hs-cTnT peak. Angiography was followed by a percutaneous coronary intervention significantly more often in patients without a second peak (85.2% of patients without and 72.1% patients with a second peak, *p* = 0.048).

**Table 1:** Baseline characteristics of patients with and without a second hs-cTnT peak.

	**All (*N* = 192)**	**No 2nd peak (*N* = 81)**	**2nd peak (*N* = 111)**	***p*-value**
Age (years)	68.0 (56.0, 76.0)	67.0 (50.0, 75.0)	69.0 (60.0, 77.8)	0.11
Male No. (%)	147 (76.6)	64 (79.0)	83 (74.8)	0.61
BMI (kg/m^2^)	27.0 (24.1, 30.2)	27.0 (24.3, 30.9)	27.2 (23.9, 29.7)	0.77
Hypertension No. (%)	151 (78.6)	62 (76.5)	89 (80.2)	0.67
Hyperlipoproteinemia No. (%)	85 (44.3)	31 (38.3)	54 (48.6)	0.20
Diabetes No. (%)	36 (18.8)	11 (13.6)	25 (22.7)	0.16
Current smoker No. (%)	57 (29.8)	18 (22.5)	39 (35.1)	0.085
History of CAD No. (%)	80 (41.7)	29 (35.8)	51 (45.9)	0.21
Angiography No. (%)	182 (94.8)	79 (97.5)	103 (92.8)	0.26
PCI No. (%)	149 (77.6)	69 (85.2)	80 (72.1)	0.048

*For continuous variables median and interquartile ranges are given. For binary variables absolute and relative frequencies are shown*.

*No., Number; BMI, body mass index; CAD, coronary artery disease; PCI, percutaneous coronary intervention*.

Regarding the composite endpoint there was no significant difference between patients with and without a second hs-cTnT peak for both unadjusted analyses [hazard ratio (HR) 0.93, 95% confidence interval (CI) 0.58–1.48, *p* = 0.75] and after adjustment for sex, age and cardiovascular risk factors (HR 0.78, CI 0.48–1.28, *p* = 0.33) (Figure 1). In comparison to hs-cTnT, CK and CK-MB showed an almost linear decrease after the index-event.

## Discussion

Our findings indicate that a second peak of hs-cTnT after MI is very common but not associated with impaired outcome compared to patients without a second peak. Our results regarding the timepoint and the height of the second peak in relation to the first peak are in line with earlier findings (3). Most of the previously published studies investigating the second hs-cTnT peak included only patients with ST-segment elevation MI (STEMI). However, we were able to confirm that a second peak was also detectable in other MI patients. Since hs-cTnT is a widely used biomarker, these novel findings are highly relevant for clinical practice. A second increase of hs-cTnT after MI can be misinterpreted as ongoing or recurrent ischemia and might lead to unnecessary coronary re-catheterization. In previous studies, a second peak after MI has only been found for hs-cTnT but not for cTnI, hs-cTnI, CK or CK-MB—irrespective of the manufacturer, the sensitivity of the assay or renal function of the patient (3). Therefore the latter might be more suitable for post-MI monitoring since a second peak of these biomarkers might indicate ischemia more accurately. Importantly, Schaaf et al. were able to find a high correlation between the second hs-cTnT peak and infarct size measured in cardiac magnetic resonance, which would somewhat disagree with our findings, since the prognosis after MI is related to the extent of the infarction (6). These investigations emphasize the need for further studies regarding the cause and impact of the second hs-cTnT peak. Several different mechanisms for the genesis of the second peak of hs-cTnT have been suggested, however, the etiology still remains unclear (3, 7). Since a second peak has only been detected for hs-cTnT but not hs-cTnI, the cause might be associated with different clearance pathways, the different molecular weight or variable fragments of the two troponins.

Our analyses and the consecutive conclusions are limited by the relatively small number of patients. Larger studies are necessary to validate the findings of this study. Also, there might be a selection bias since troponin was not measured systematically in all included patients but as indicated by the treating physician. Therefore, a second hs-cTnT peak might have been missed in some patients. The cut-off of 15%, which was required for an increase of troponin to be defined as a second peak in this study, was chosen to exclude a random variability of two consecutive hs-cTnT measurements. However, this cut-off value should be validated in a larger study. Lastly, due to the limited number of patients in this study, we cannot make conclusions on whether the second peak has other characteristics or a different clinical value in certain types of MI.

In summary, the clinical value of hs-cTnT measurements after MI might be limited due a frequently occurring second peak, which was not associated with impaired outcome in our analyses. Prospective studies are needed to further evaluate the role of hs-cTnT after MI.

## Data Availability Statement

The raw data supporting the conclusions of this article will be made available by the authors, without undue reservation.

## Ethics Statement

The studies involving human participants were reviewed and approved by the local ethics committee. The patients/participants provided their written informed consent to participate in this study.

## Author Contributions

TH, JN, and DW made significant contributions to the conception and design of the study. AG contributed to the analysis of the data. TH and JN drafted the manuscript. All authors critically revised the manuscript for intellectual content, approved it for submission, and were involved in the acquisition and interpretation of the data.

## Funding

This work was supported by the German Center of Cardiovascular Research (DZHK) and with an unrestricted grant by Abbott Diagnostics. JN was recipient of a research fellowship by the Deutsche Forschungsgemeinschaft (DFG).

## Conflict of Interest

SB has received honoraria from Abbott Diagnostics, Siemens, Thermo Fisher, and Roche Diagnostics and is a consultant for Thermo Fisher; DW reports personal fees from Bayer, Boehringer-Ingelheim, Berlin Chemie, Astra Zeneca, Biotronik and Novartis. The remaining authors declare that the research was conducted in the absence of any commercial or financial relationships that could be construed as a potential conflict of interest.

## Publisher's Note

All claims expressed in this article are solely those of the authors and do not necessarily represent those of their affiliated organizations, or those of the publisher, the editors and the reviewers. Any product that may be evaluated in this article, or claim that may be made by its manufacturer, is not guaranteed or endorsed by the publisher.
